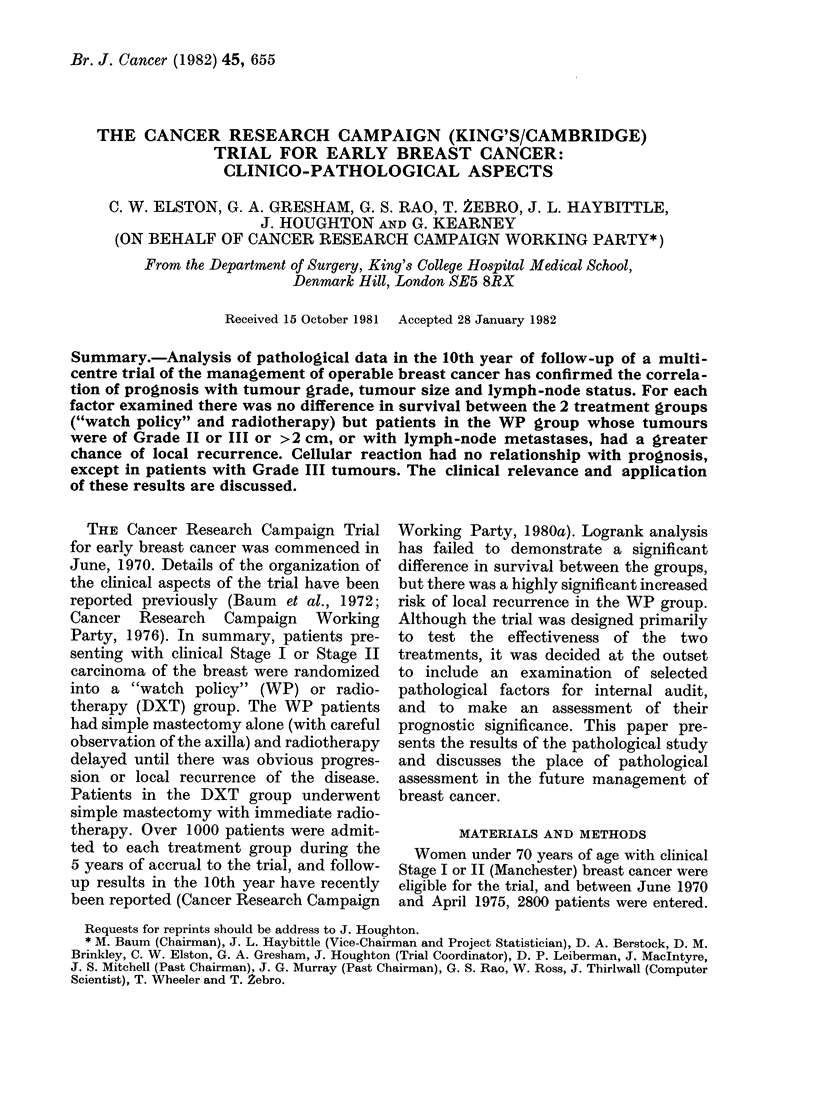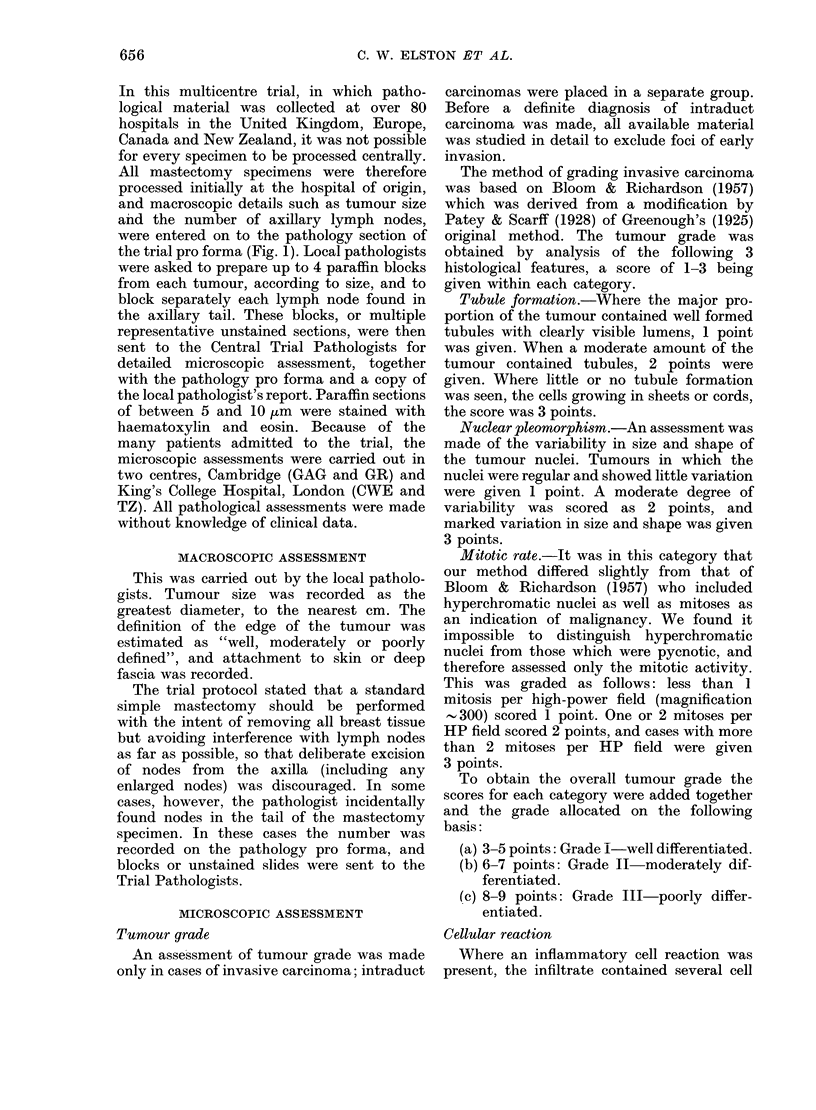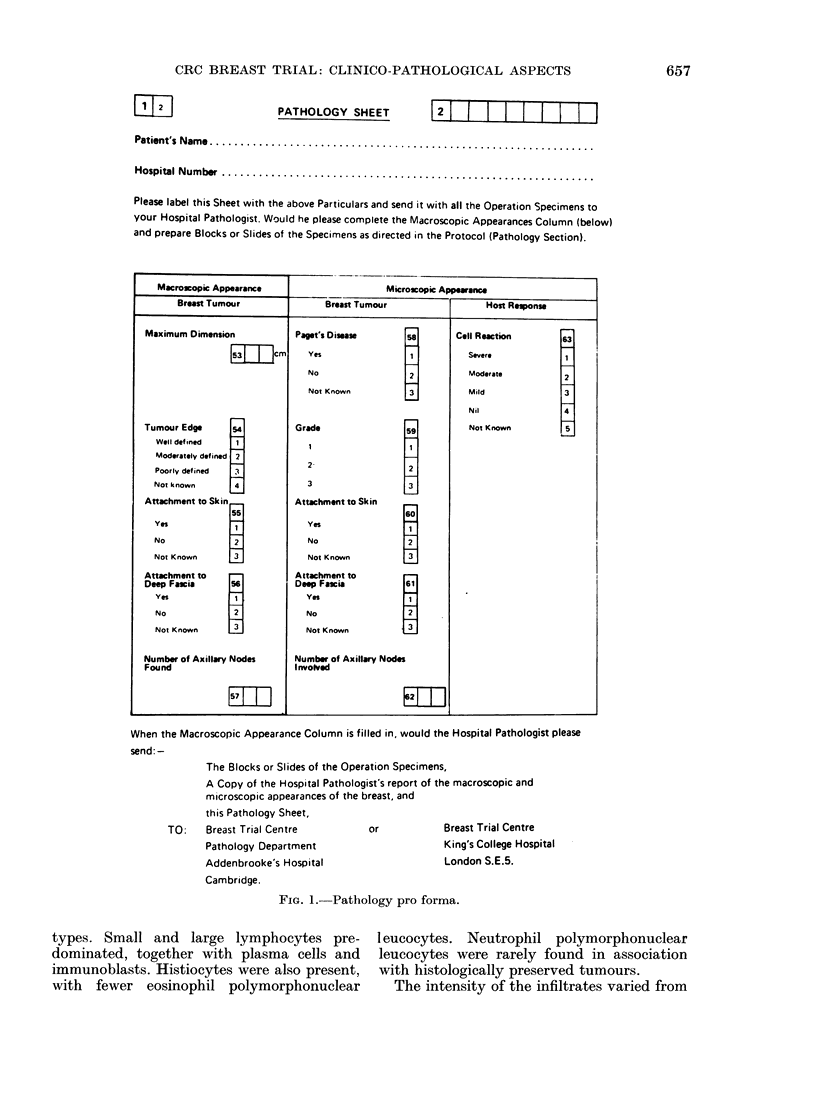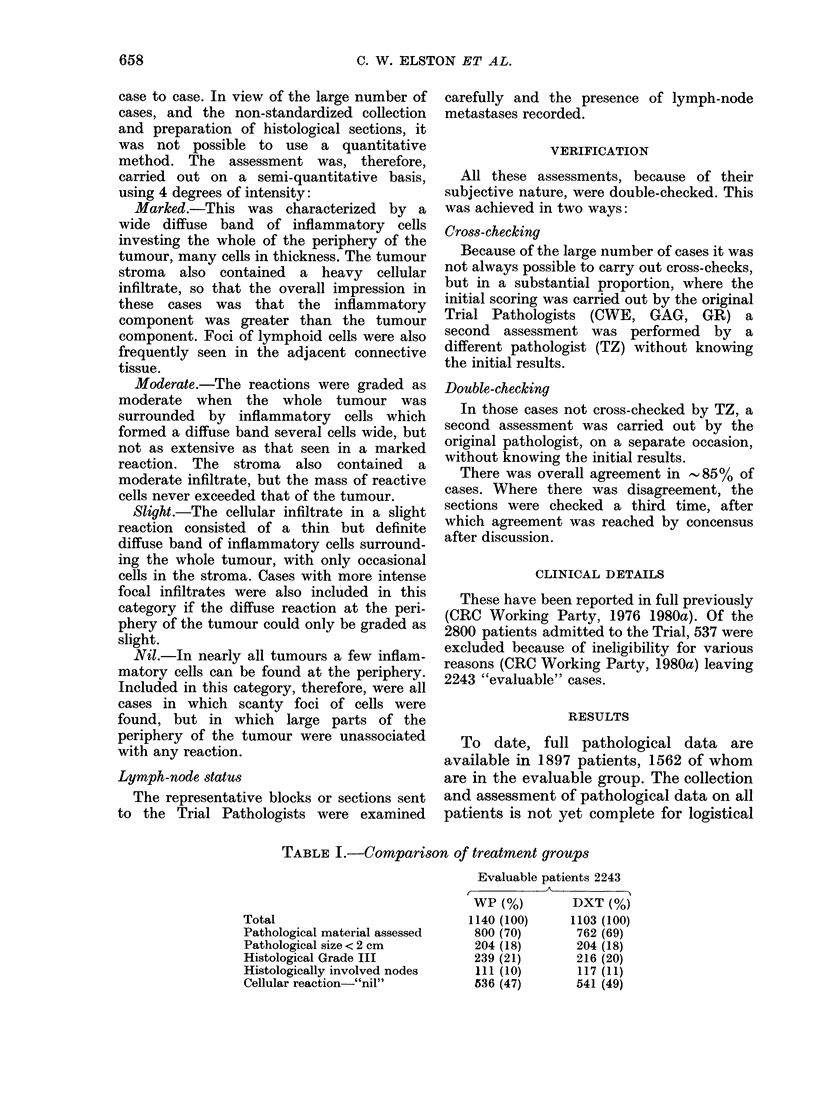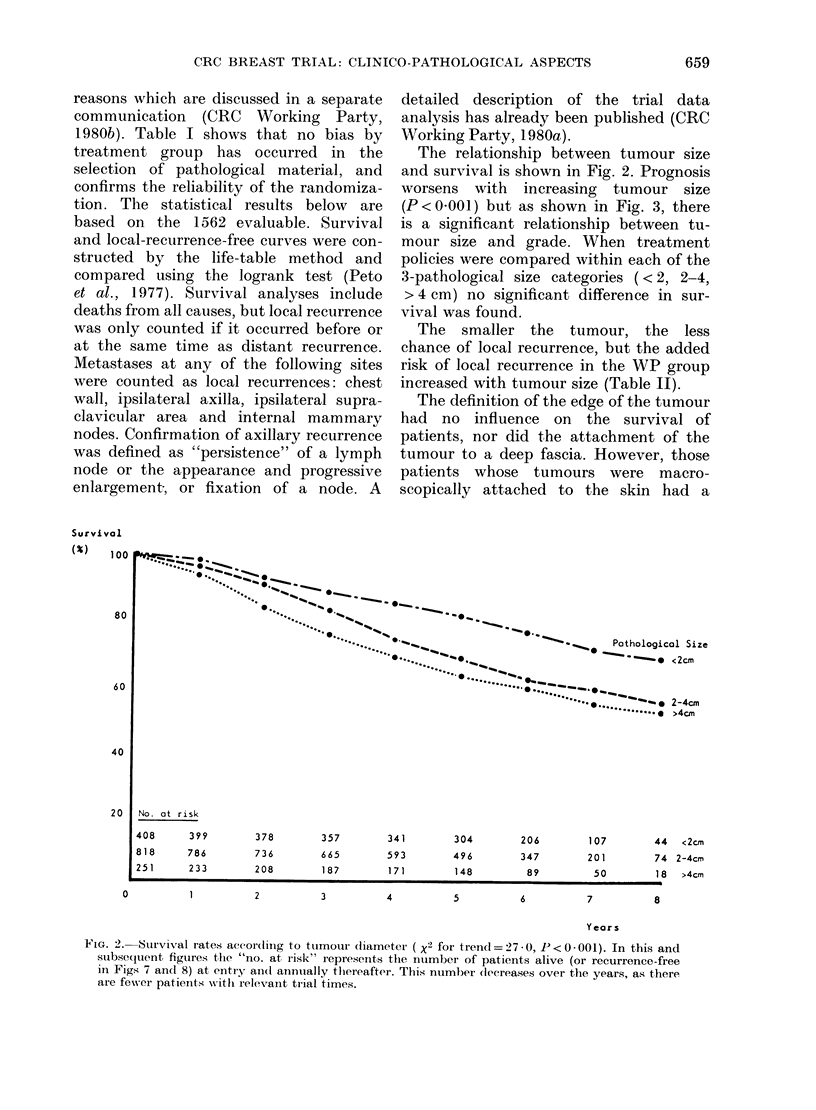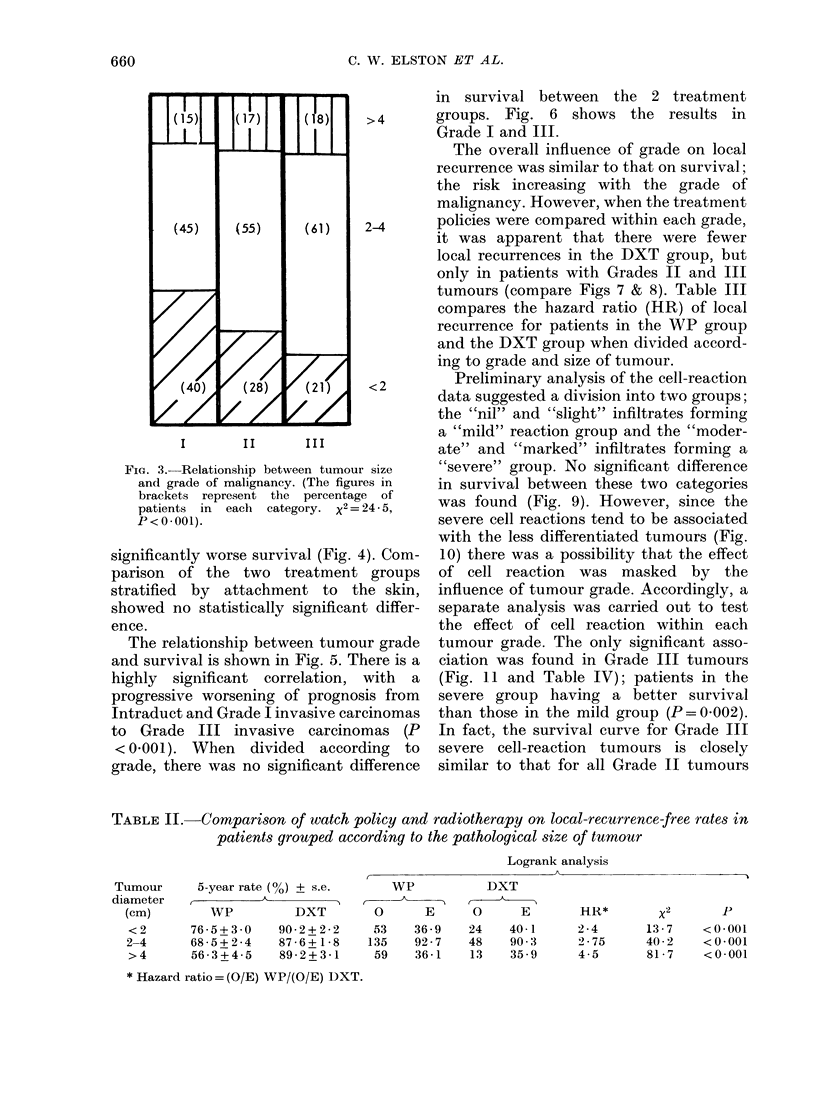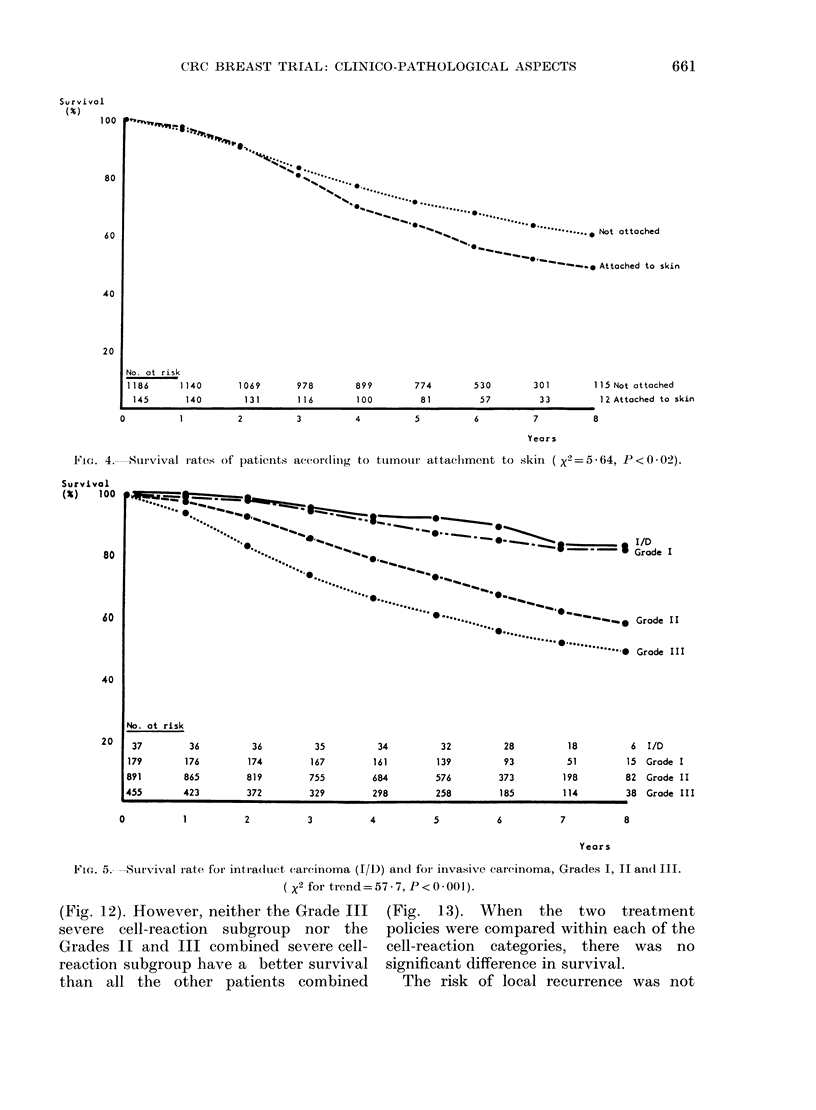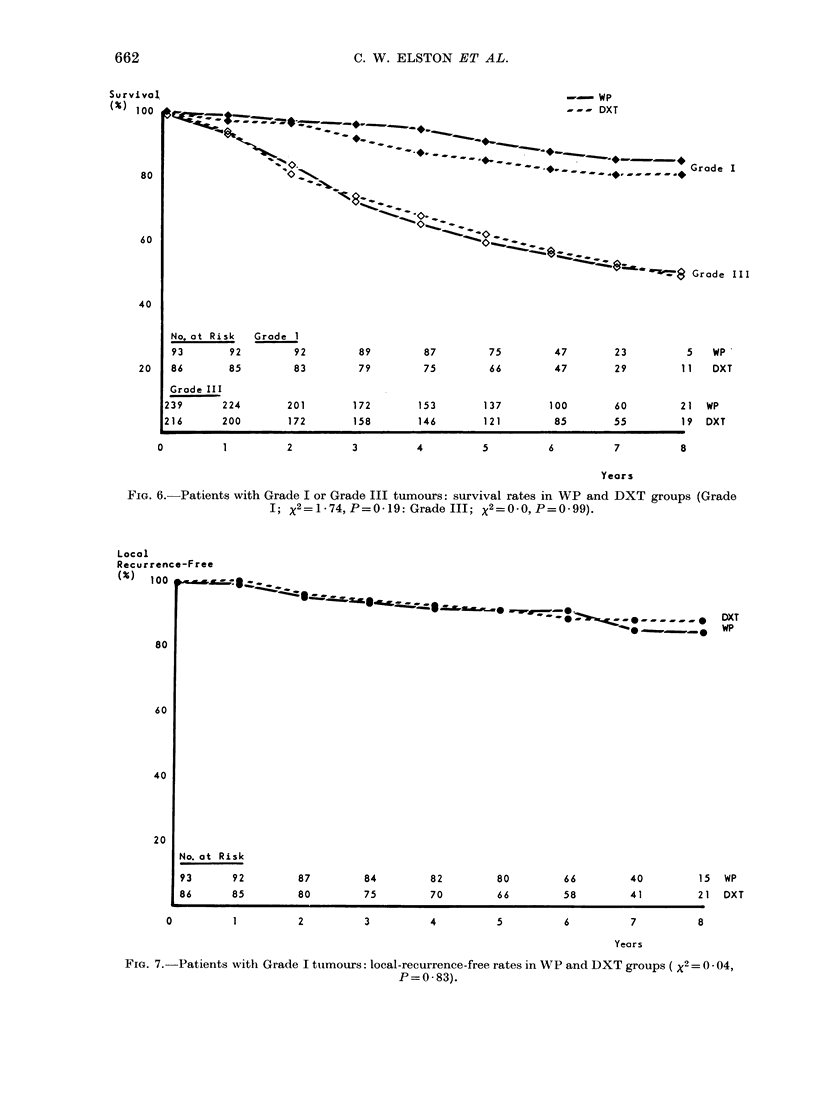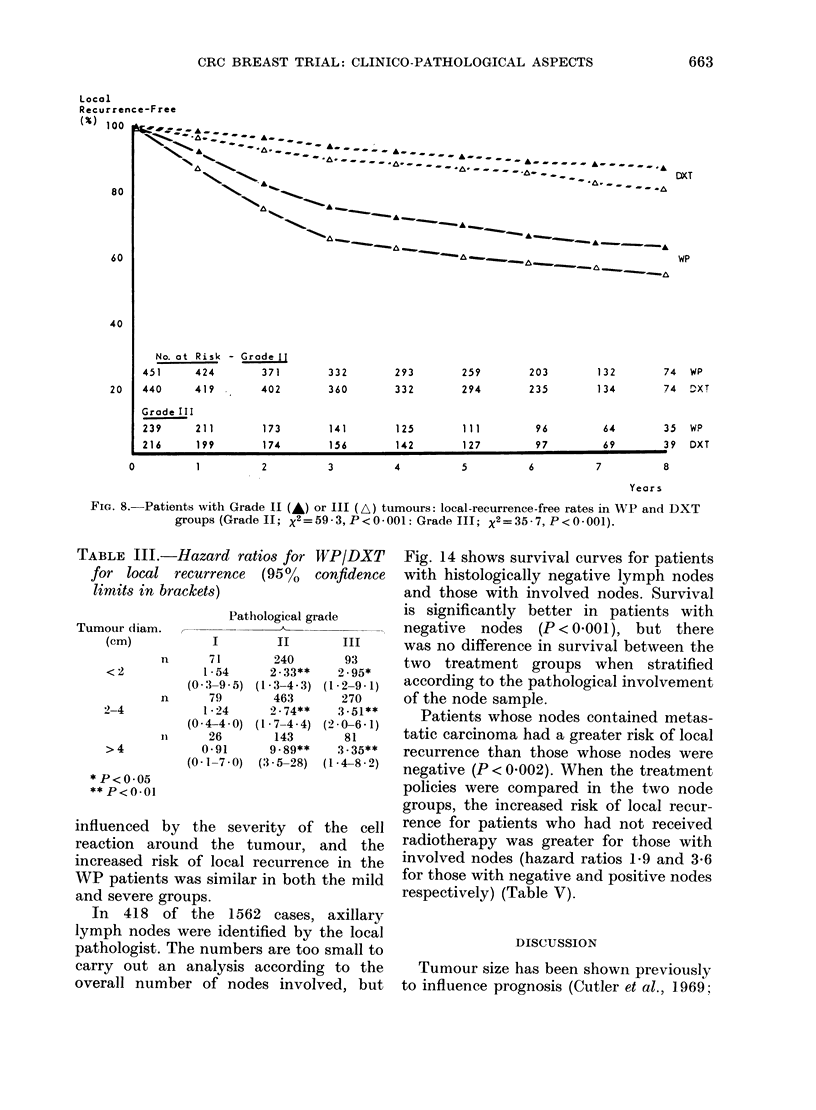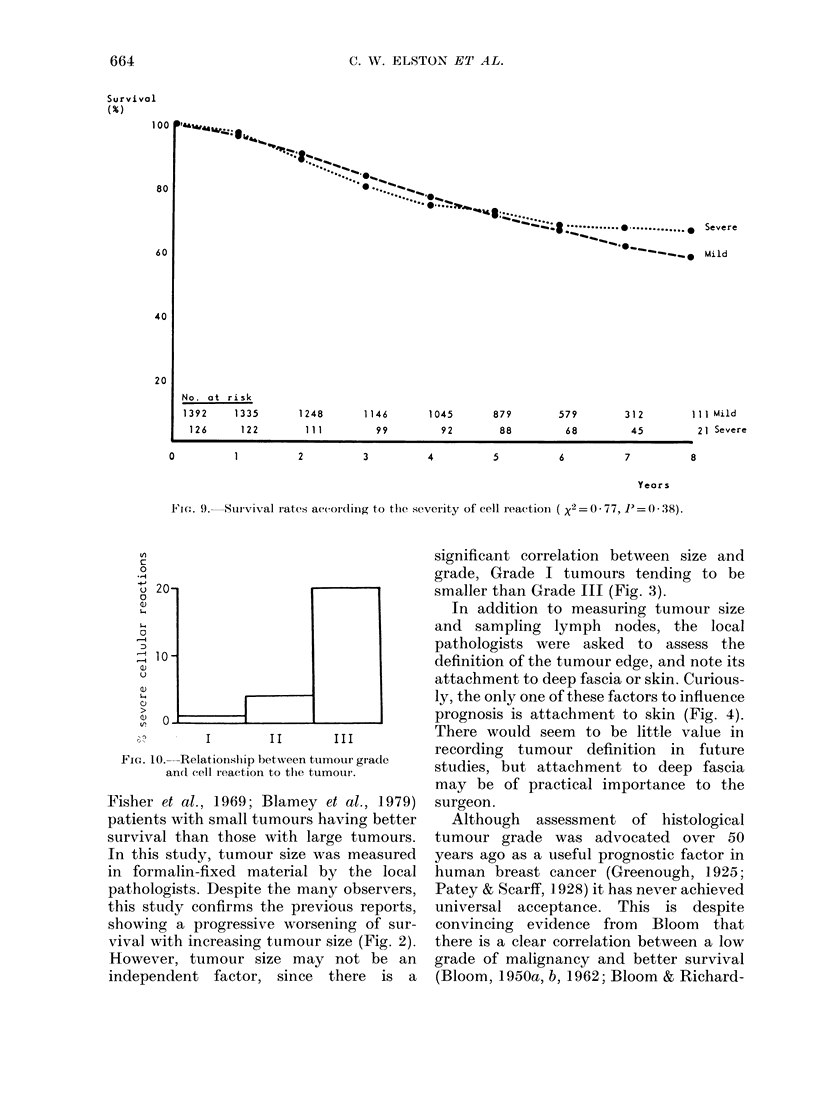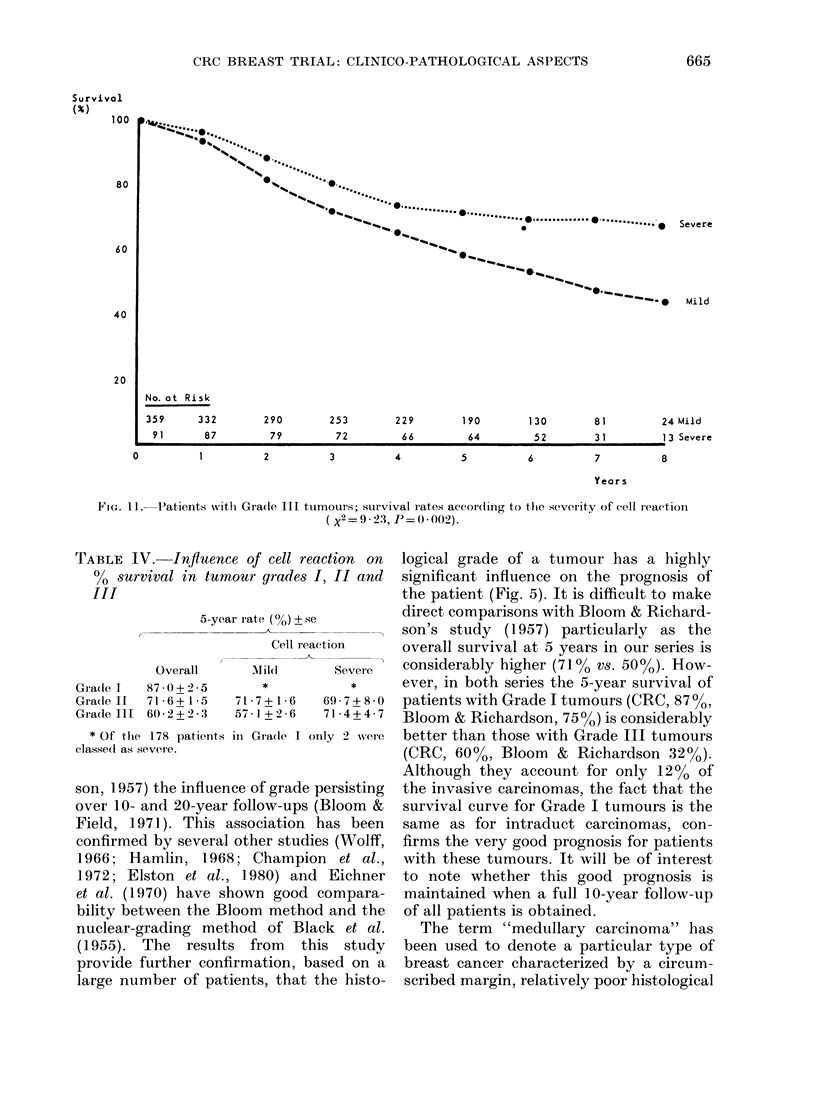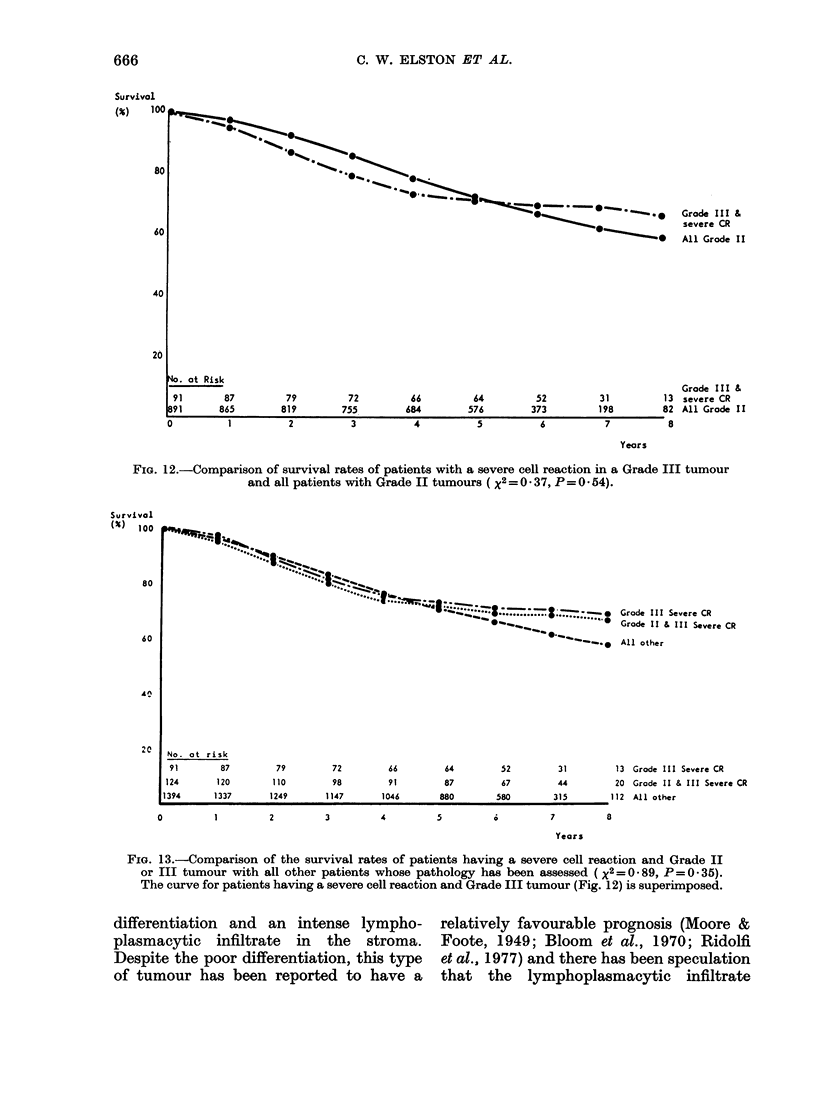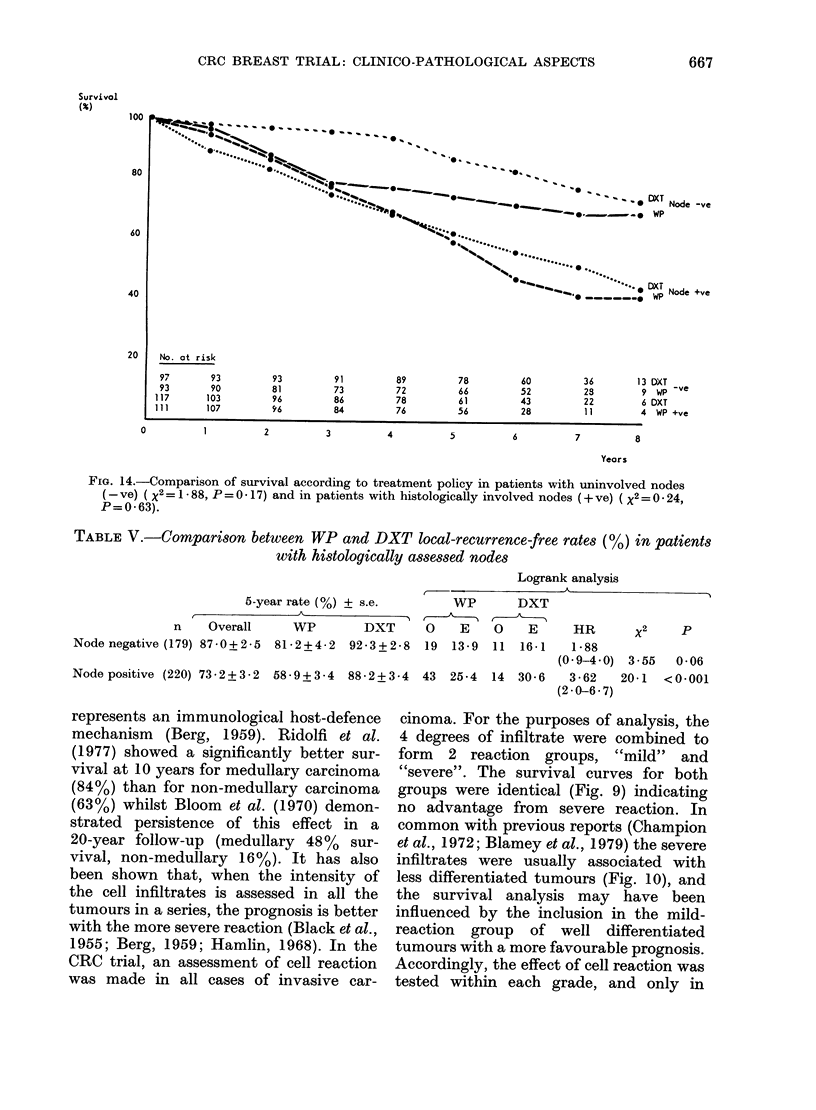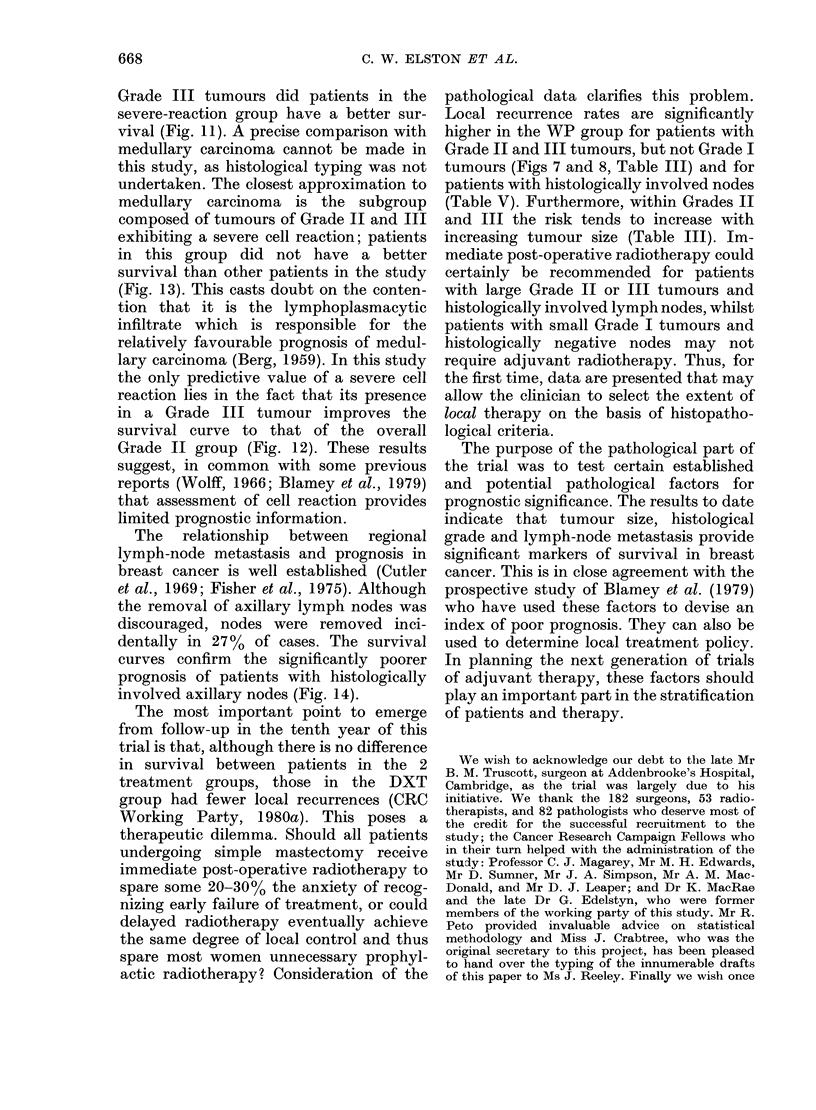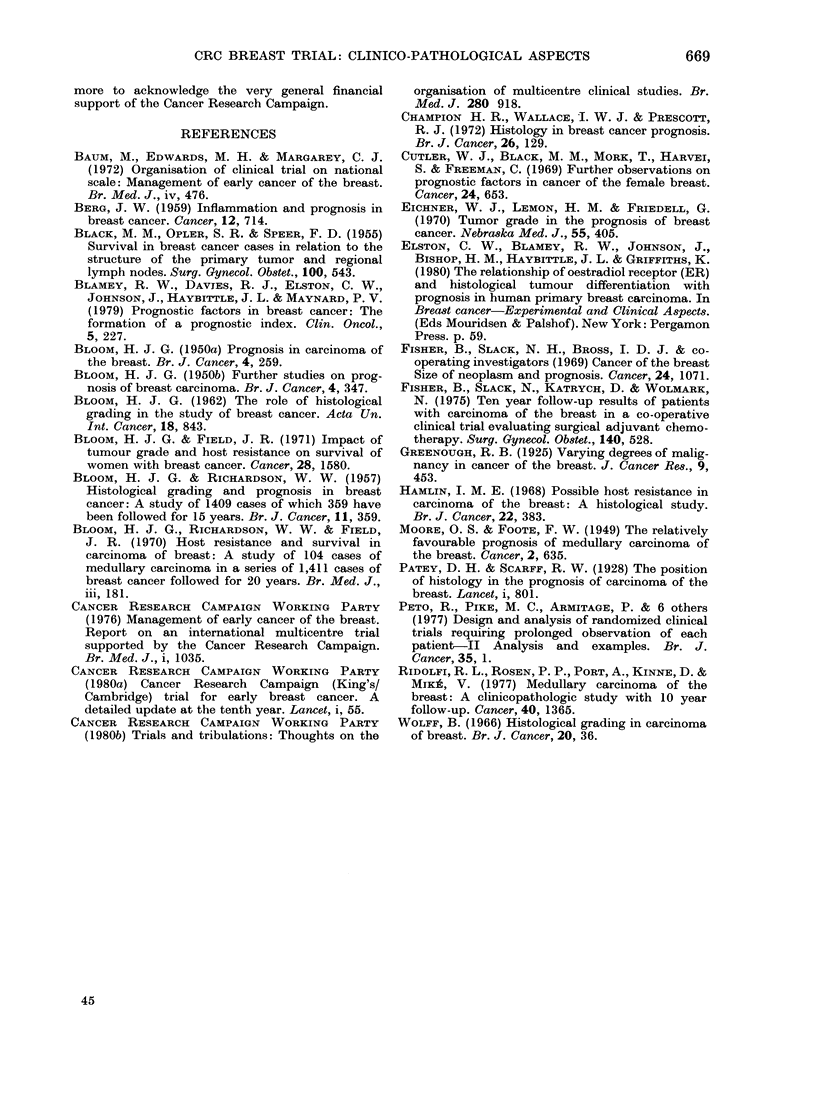# The cancer research campaign (King's/Cambridge trial for early breast cancer: clinico-pathological aspects.

**DOI:** 10.1038/bjc.1982.106

**Published:** 1982-05

**Authors:** C. W. Elston, G. A. Gresham, G. S. Rao, T. Zebro, J. L. Haybittle, J. Houghton, G. Kearney

## Abstract

Analysis of pathological data in the 10th year of follow-up of a multicentre trial of the management of operable breast cancer has confirmed the correlation of prognosis with tumour grade, tumour size and lymph-node status. For each factor examined there was no difference in survival between the 2 treatment groups ("watch policy" and radiotherapy) but patients in the WP group whose tumours were of Grade II or III or greater than 2 cm, or with lymph-node metastases, had a greater chance of local recurrence. Cellular reaction had no relationship with prognosis, except in patients with Grade III tumours. The clinical relevance and application of these results are discussed.


*Br. J. Cancer* (1982) **45**, 655

### THE CANCER RESEARCH CAMPAIGN (KING'S/CAMBRIDGE) TRIAL FOR EARLY BREAST CANCER: CLINICO-PATHOLOGICAL ASPECTS

C. W. ELSTON, G. A. GRESHAM, G. S. RAO, T. ŻEBRO, J. L. HAYBITTLE, J. HOUGHTON AND G. KEARNEY
(ON BEHALF OF CANCER RESEARCH CAMPAIGN WORKING PARTY*)

*From the Department of Surgery, King's College Hospital Medical School, Denmark Hill, London SE5 8RX*

Received 15 October 1981 Accepted 28 January 1982

**Summary.—Analysis of pathological data in the 10th year of follow-up of a multicentre trial of the management of operable breast cancer has confirmed the correlation of prognosis with tumour grade, tumour size and lymph-node status. For each factor examined there was no difference in survival between the 2 treatment groups ("watch policy" and radiotherapy) but patients in the WP group whose tumours were of Grade II or III or >2 cm, or with lymph-node metastases, had a greater chance of local recurrence. Cellular reaction had no relationship with prognosis, except in patients with Grade III tumours. The clinical relevance and application of these results are discussed.**

THE Cancer Research Campaign Trial for early breast cancer was commenced in June, 1970. Details of the organization of the clinical aspects of the trial have been reported previously (Baum *et al.*, 1972; Cancer Research Campaign Working Party, 1976). In summary, patients presenting with clinical Stage I or Stage II carcinoma of the breast were randomized into a "watch policy" (WP) or radiotherapy (DXT) group. The WP patients had simple mastectomy alone (with careful observation of the axilla) and radiotherapy delayed until there was obvious progression or local recurrence of the disease. Patients in the DXT group underwent simple mastectomy with immediate radiotherapy. Over 1000 patients were admitted to each treatment group during the 5 years of accrual to the trial, and follow-up results in the 10th year have recently been reported (Cancer Research Campaign Working Party, 1980*a*). Logrank analysis has failed to demonstrate a significant difference in survival between the groups, but there was a highly significant increased risk of local recurrence in the WP group. Although the trial was designed primarily to test the effectiveness of the two treatments, it was decided at the outset to include an examination of selected pathological factors for internal audit, and to make an assessment of their prognostic significance. This paper presents the results of the pathological study and discusses the place of pathological assessment in the future management of breast cancer.

### MATERIALS AND METHODS

Women under 70 years of age with clinical Stage I or II (Manchester) breast cancer were eligible for the trial, and between June 1970 and April 1975, 2800 patients were entered.

Requests for reprints should be address to J. Houghton.

* M. Baum (Chairman), J. L. Haybittle (Vice-Chairman and Project Statistician), D. A. Berstock, D. M. Brinkley, C. W. Elston, G. A. Gresham, J. Houghton (Trial Coordinator), D. P. Leiberman, J. MacIntyre, J. S. Mitchell (Past Chairman), J. G. Murray (Past Chairman), G. S. Rao, W. Ross, J. Thirlwall (Computer Scientist), T. Wheeler and T. Żebro.

In this multicentre trial, in which pathological material was collected at over 80 hospitals in the United Kingdom, Europe, Canada and New Zealand, it was not possible for every specimen to be processed centrally. All mastectomy specimens were therefore processed initially at the hospital of origin, and macroscopic details such as tumour size and the number of axillary lymph nodes, were entered on to the pathology section of the trial pro forma (Fig. 1). Local pathologists were asked to prepare up to 4 paraffin blocks from each tumour, according to size, and to block separately each lymph node found in the axillary tail. These blocks, or multiple representative unstained sections, were then sent to the Central Trial Pathologists for detailed microscopic assessment, together with the pathology pro forma and a copy of the local pathologist's report. Paraffin sections of between 5 and 10 $\mu$m were stained with haematoxylin and eosin. Because of the many patients admitted to the trial, the microscopic assessments were carried out in two centres, Cambridge (GAG and GR) and King's College Hospital, London (CWE and TZ). All pathological assessments were made without knowledge of clinical data.

### MACROSCOPIC ASSESSMENT

This was carried out by the local pathologists. Tumour size was recorded as the greatest diameter, to the nearest cm. The definition of the edge of the tumour was estimated as "well, moderately or poorly defined", and attachment to skin or deep fascia was recorded.

The trial protocol stated that a standard simple mastectomy should be performed with the intent of removing all breast tissue but avoiding interference with lymph nodes as far as possible, so that deliberate excision of nodes from the axilla (including any enlarged nodes) was discouraged. In some cases, however, the pathologist incidentally found nodes in the tail of the mastectomy specimen. In these cases the number was recorded on the pathology pro forma, and blocks or unstained slides were sent to the Trial Pathologists.

### MICROSCOPIC ASSESSMENT

### *Tumour grade*

An assessment of tumour grade was made only in cases of invasive carcinoma; intraduct carcinomas were placed in a separate group. Before a definite diagnosis of intraduct carcinoma was made, all available material was studied in detail to exclude foci of early invasion.

The method of grading invasive carcinoma was based on Bloom & Richardson (1957) which was derived from a modification by Patey & Scarff (1928) of Greenough's (1925) original method. The tumour grade was obtained by analysis of the following 3 histological features, a score of 1–3 being given within each category.

*Tubule formation.*—Where the major proportion of the tumour contained well formed tubules with clearly visible lumens, 1 point was given. When a moderate amount of the tumour contained tubules, 2 points were given. Where little or no tubule formation was seen, the cells growing in sheets or cords, the score was 3 points.

*Nuclear pleomorphism.*—An assessment was made of the variability in size and shape of the tumour nuclei. Tumours in which the nuclei were regular and showed little variation were given 1 point. A moderate degree of variability was scored as 2 points, and marked variation in size and shape was given 3 points.

*Mitotic rate.*—It was in this category that our method differed slightly from that of Bloom & Richardson (1957) who included hyperchromatic nuclei as well as mitoses as an indication of malignancy. We found it impossible to distinguish hyperchromatic nuclei from those which were pycnotic, and therefore assessed only the mitotic activity. This was graded as follows: less than 1 mitosis per high-power field (magnification $\sim$300) scored 1 point. One or 2 mitoses per HP field scored 2 points, and cases with more than 2 mitoses per HP field were given 3 points.

To obtain the overall tumour grade the scores for each category were added together and the grade allocated on the following basis:

(a) 3–5 points: Grade I—well differentiated.
(b) 6–7 points: Grade II—moderately differentiated.
(c) 8–9 points: Grade III—poorly differentiated.

### *Cellular reaction*

Where an inflammatory cell reaction was present, the infiltrate contained several cell

1 2

**PATHOLOGY SHEET** 2

**Patient's Name**..........................................................

**Hospital Number**..........................................................

Please label this Sheet with the above Particulars and send it with all the Operation Specimens to your Hospital Pathologist. Would he please complete the Macroscopic Appearances Column (below) and prepare Blocks or Slides of the Specimens as directed in the Protocol (Pathology Section).

| Macroscopic Appearance | Microscopic Appearance | |
|---|---|---|
| **Breast Tumour** | **Breast Tumour** | **Host Response** |
| **Maximum Dimension** 53 cm | **Paget's Disease** 58 | **Cell Reaction** 63 |
| | Yes 1 | Severe 1 |
| | No 2 | Moderate 2 |
| | Not Known 3 | Mild 3 |
| | | Nil 4 |
| **Tumour Edge** 54 | **Grade** 59 | Not Known 5 |
| Well defined 1 | 1 1 | |
| Moderately defined 2 | 2 2 | |
| Poorly defined 3 | 3 3 | |
| Not known 4 | | |
| **Attachment to Skin** 55 | **Attachment to Skin** 60 | |
| Yes 1 | Yes 1 | |
| No 2 | No 2 | |
| Not Known 3 | Not Known 3 | |
| **Attachment to Deep Fascia** 56 | **Attachment to Deep Fascia** 61 | |
| Yes 1 | Yes 1 | |
| No 2 | No 2 | |
| Not Known 3 | Not Known 3 | |
| **Number of Axillary Nodes Found** 57 | **Number of Axillary Nodes Involved** 62 | |

When the Macroscopic Appearance Column is filled in, would the Hospital Pathologist please send:–

The Blocks or Slides of the Operation Specimens,

A Copy of the Hospital Pathologist's report of the macroscopic and microscopic appearances of the breast, and this Pathology Sheet,

TO: Breast Trial Centre<br>Pathology Department<br>Addenbrooke's Hospital<br>Cambridge.

or

Breast Trial Centre<br>King's College Hospital<br>London S.E.5.

FIG. 1.—Pathology pro forma.

types. Small and large lymphocytes predominated, together with plasma cells and immunoblasts. Histiocytes were also present, with fewer eosinophil polymorphonuclear leucocytes. Neutrophil polymorphonuclear leucocytes were rarely found in association with histologically preserved tumours.

The intensity of the infiltrates varied from case to case. In view of the large number of cases, and the non-standardized collection and preparation of histological sections, it was not possible to use a quantitative method. The assessment was, therefore, carried out on a semi-quantitative basis, using 4 degrees of intensity:

*Marked.*—This was characterized by a wide diffuse band of inflammatory cells investing the whole of the periphery of the tumour, many cells in thickness. The tumour stroma also contained a heavy cellular infiltrate, so that the overall impression in these cases was that the inflammatory component was greater than the tumour component. Foci of lymphoid cells were also frequently seen in the adjacent connective tissue.

*Moderate.*—The reactions were graded as moderate when the whole tumour was surrounded by inflammatory cells which formed a diffuse band several cells wide, but not as extensive as that seen in a marked reaction. The stroma also contained a moderate infiltrate, but the mass of reactive cells never exceeded that of the tumour.

*Slight.*—The cellular infiltrate in a slight reaction consisted of a thin but definite diffuse band of inflammatory cells surrounding the whole tumour, with only occasional cells in the stroma. Cases with more intense focal infiltrates were also included in this category if the diffuse reaction at the periphery of the tumour could only be graded as slight.

*Nil.*—In nearly all tumours a few inflammatory cells can be found at the periphery. Included in this category, therefore, were all cases in which scanty foci of cells were found, but in which large parts of the periphery of the tumour were unassociated with any reaction.

### *Lymph-node status*

The representative blocks or sections sent to the Trial Pathologists were examined carefully and the presence of lymph-node metastases recorded.

### VERIFICATION

All these assessments, because of their subjective nature, were double-checked. This was achieved in two ways:

### *Cross-checking*

Because of the large number of cases it was not always possible to carry out cross-checks, but in a substantial proportion, where the initial scoring was carried out by the original Trial Pathologists (CWE, GAG, GR) a second assessment was performed by a different pathologist (TZ) without knowing the initial results.

### *Double-checking*

In those cases not cross-checked by TZ, a second assessment was carried out by the original pathologist, on a separate occasion, without knowing the initial results.

There was overall agreement in $\sim 85\%$ of cases. Where there was disagreement, the sections were checked a third time, after which agreement was reached by concensus after discussion.

### CLINICAL DETAILS

These have been reported in full previously (CRC Working Party, 1976 1980*a*). Of the 2800 patients admitted to the Trial, 537 were excluded because of ineligibility for various reasons (CRC Working Party, 1980*a*) leaving 2243 "evaluable" cases.

### RESULTS

To date, full pathological data are available in 1897 patients, 1562 of whom are in the evaluable group. The collection and assessment of pathological data on all patients is not yet complete for logistical

TABLE I.—*Comparison of treatment groups*

| | Evaluable patients 2243 | |
|---|---|---|
| | WP (%) | DXT (%) |
| Total | 1140 (100) | 1103 (100) |
| Pathological material assessed | 800 (70) | 762 (69) |
| Pathological size < 2 cm | 204 (18) | 204 (18) |
| Histological Grade III | 239 (21) | 216 (20) |
| Histologically involved nodes | 111 (10) | 117 (11) |
| Cellular reaction—"nil" | 536 (47) | 541 (49) |

reasons which are discussed in a separate communication (CRC Working Party, 1980*b*). Table I shows that no bias by treatment group has occurred in the selection of pathological material, and confirms the reliability of the randomization. The statistical results below are based on the 1562 evaluable. Survival and local-recurrence-free curves were constructed by the life-table method and compared using the logrank test (Peto *et al.*, 1977). Survival analyses include deaths from all causes, but local recurrence was only counted if it occurred before or at the same time as distant recurrence. Metastases at any of the following sites were counted as local recurrences: chest wall, ipsilateral axilla, ipsilateral supraclavicular area and internal mammary nodes. Confirmation of axillary recurrence was defined as "persistence" of a lymph node or the appearance and progressive enlargement, or fixation of a node. A detailed description of the trial data analysis has already been published (CRC Working Party, 1980*a*).

The relationship between tumour size and survival is shown in Fig. 2. Prognosis worsens with increasing tumour size ($P<0{\cdot}001$) but as shown in Fig. 3, there is a significant relationship between tumour size and grade. When treatment policies were compared within each of the 3-pathological size categories ($<2$, 2–4, $>4$ cm) no significant difference in survival was found.

The smaller the tumour, the less chance of local recurrence, but the added risk of local recurrence in the WP group increased with tumour size (Table II).

The definition of the edge of the tumour had no influence on the survival of patients, nor did the attachment of the tumour to a deep fascia. However, those patients whose tumours were macroscopically attached to the skin had a

FIG. 2.—Survival rates according to tumour diameter ($\chi^2$ for trend $= 27{\cdot}0$, $P<0{\cdot}001$). In this and subsequent figures the "no. at risk" represents the number of patients alive (or recurrence-free in Figs 7 and 8) at entry and annually thereafter. This number decreases over the years, as there are fewer patients with relevant trial times.

FIG. 3.—Relationship between tumour size and grade of malignancy. (The figures in brackets represent the percentage of patients in each category. $\chi^2 = 24 \cdot 5$, $P < 0 \cdot 001$).

significantly worse survival (Fig. 4). Comparison of the two treatment groups stratified by attachment to the skin, showed no statistically significant difference.

The relationship between tumour grade and survival is shown in Fig. 5. There is a highly significant correlation, with a progressive worsening of prognosis from Intraduct and Grade I invasive carcinomas to Grade III invasive carcinomas ($P < 0{\cdot}001$). When divided according to grade, there was no significant difference in survival between the 2 treatment groups. Fig. 6 shows the results in Grade I and III.

The overall influence of grade on local recurrence was similar to that on survival; the risk increasing with the grade of malignancy. However, when the treatment policies were compared within each grade, it was apparent that there were fewer local recurrences in the DXT group, but only in patients with Grades II and III tumours (compare Figs 7 & 8). Table III compares the hazard ratio (HR) of local recurrence for patients in the WP group and the DXT group when divided according to grade and size of tumour.

Preliminary analysis of the cell-reaction data suggested a division into two groups; the "nil" and "slight" infiltrates forming a "mild" reaction group and the "moderate" and "marked" infiltrates forming a "severe" group. No significant difference in survival between these two categories was found (Fig. 9). However, since the severe cell reactions tend to be associated with the less differentiated tumours (Fig. 10) there was a possibility that the effect of cell reaction was masked by the influence of tumour grade. Accordingly, a separate analysis was carried out to test the effect of cell reaction within each tumour grade. The only significant association was found in Grade III tumours (Fig. 11 and Table IV); patients in the severe group having a better survival than those in the mild group ($P = 0{\cdot}002$). In fact, the survival curve for Grade III severe cell-reaction tumours is closely similar to that for all Grade II tumours

TABLE II.—*Comparison of watch policy and radiotherapy on local-recurrence-free rates in patients grouped according to the pathological size of tumour*

| Tumour diameter (cm) | 5-year rate (%) ± s.e. | | Logrank analysis | | | | | | |
|---|---|---|---|---|---|---|---|---|---|
| | | | WP | | DXT | | | | |
| | WP | DXT | O | E | O | E | HR* | $\chi^2$ | $P$ |
| < 2 | 76·5±3·0 | 90·2±2·2 | 53 | 36·9 | 24 | 40·1 | 2·4 | 13·7 | < 0·001 |
| 2–4 | 68·5±2·4 | 87·6±1·8 | 135 | 92·7 | 48 | 90·3 | 2·75 | 40·2 | < 0·001 |
| > 4 | 56·3±4·5 | 89·2±3·1 | 59 | 36·1 | 13 | 35·9 | 4·5 | 81·7 | < 0·001 |

* Hazard ratio = (O/E) WP/(O/E) DXT.

FIG. 4.—Survival rates of patients according to tumour attachment to skin ($\chi^2 = 5 \cdot 64$, $P < 0 \cdot 02$).

FIG. 5.—Survival rate for intraduct carcinoma (I/D) and for invasive carcinoma, Grades I, II and III. ($\chi^2$ for trend $= 57 \cdot 7$, $P < 0 \cdot 001$).

(Fig. 12). However, neither the Grade III severe cell-reaction subgroup nor the Grades II and III combined severe cell-reaction subgroup have a better survival than all the other patients combined (Fig. 13). When the two treatment policies were compared within each of the cell-reaction categories, there was no significant difference in survival.

The risk of local recurrence was not

FIG. 6.—Patients with Grade I or Grade III tumours: survival rates in WP and DXT groups (Grade I; $\chi^2 = 1 \cdot 74$, $P = 0 \cdot 19$: Grade III; $\chi^2 = 0 \cdot 0$, $P = 0 \cdot 99$).

FIG. 7.—Patients with Grade I tumours: local-recurrence-free rates in WP and DXT groups ( $\chi^2 = 0 \cdot 04$, $P = 0 \cdot 83$).

FIG. 8.—Patients with Grade II (▲) or III (△) tumours: local-recurrence-free rates in WP and DXT groups (Grade II; $\chi^2 = 59 \cdot 3$, $P < 0 \cdot 001$: Grade III; $\chi^2 = 35 \cdot 7$, $P < 0 \cdot 001$).

TABLE III.—*Hazard ratios for WP/DXT for local recurrence (95% confidence limits in brackets)*

| Tumour diam. (cm) | | Pathological grade I | II | III |
|---|---|---|---|---|
| | n | 71 | 240 | 93 |
| <2 | | 1·54 | 2·33** | 2·95* |
| | | (0·3–9·5) | (1·3–4·3) | (1·2–9·1) |
| | n | 79 | 463 | 270 |
| 2–4 | | 1·24 | 2·74** | 3·51** |
| | | (0·4–4·0) | (1·7–4·4) | (2·0–6·1) |
| | n | 26 | 143 | 81 |
| >4 | | 0·91 | 9·89** | 3·35** |
| | | (0·1–7·0) | (3·5–28) | (1·4–8·2) |

* $P < 0 \cdot 05$
** $P < 0 \cdot 01$

influenced by the severity of the cell reaction around the tumour, and the increased risk of local recurrence in the WP patients was similar in both the mild and severe groups.

In 418 of the 1562 cases, axillary lymph nodes were identified by the local pathologist. The numbers are too small to carry out an analysis according to the overall number of nodes involved, but Fig. 14 shows survival curves for patients with histologically negative lymph nodes and those with involved nodes. Survival is significantly better in patients with negative nodes ($P < 0 \cdot 001$), but there was no difference in survival between the two treatment groups when stratified according to the pathological involvement of the node sample.

Patients whose nodes contained metastatic carcinoma had a greater risk of local recurrence than those whose nodes were negative ($P < 0 \cdot 002$). When the treatment policies were compared in the two node groups, the increased risk of local recurrence for patients who had not received radiotherapy was greater for those with involved nodes (hazard ratios 1·9 and 3·6 for those with negative and positive nodes respectively) (Table V).

### DISCUSSION

Tumour size has been shown previously to influence prognosis (Cutler *et al.*, 1969;

FIG. 9.—Survival rates according to the severity of cell reaction ( $\chi^2=0\cdot77, P=0\cdot38$).

FIG. 10.—Relationship between tumour grade and cell reaction to the tumour.

Fisher *et al.*, 1969; Blamey *et al.*, 1979) patients with small tumours having better survival than those with large tumours. In this study, tumour size was measured in formalin-fixed material by the local pathologists. Despite the many observers, this study confirms the previous reports, showing a progressive worsening of survival with increasing tumour size (Fig. 2). However, tumour size may not be an independent factor, since there is a significant correlation between size and grade, Grade I tumours tending to be smaller than Grade III (Fig. 3).

In addition to measuring tumour size and sampling lymph nodes, the local pathologists were asked to assess the definition of the tumour edge, and note its attachment to deep fascia or skin. Curiously, the only one of these factors to influence prognosis is attachment to skin (Fig. 4). There would seem to be little value in recording tumour definition in future studies, but attachment to deep fascia may be of practical importance to the surgeon.

Although assessment of histological tumour grade was advocated over 50 years ago as a useful prognostic factor in human breast cancer (Greenough, 1925; Patey & Scarff, 1928) it has never achieved universal acceptance. This is despite convincing evidence from Bloom that there is a clear correlation between a low grade of malignancy and better survival (Bloom, 1950*a*, *b*, 1962; Bloom & Richard-

FIG. 11.—Patients with Grade III tumours; survival rates according to the severity of cell reaction ($\chi^2 = 9 \cdot 23$, $P = 0 \cdot 002$).

TABLE IV.—*Influence of cell reaction on % survival in tumour grades I, II and III*

| | 5-year rate (%) ± se | | |
|---|---|---|---|
| | | Cell reaction | |
| | Overall | Mild | Severe |
| Grade I | 87·0 ± 2·5 | * | * |
| Grade II | 71·6 ± 1·5 | 71·7 ± 1·6 | 69·7 ± 8·0 |
| Grade III | 60·2 ± 2·3 | 57·1 ± 2·6 | 71·4 ± 4·7 |

* Of the 178 patients in Grade I only 2 were classed as severe.

son, 1957) the influence of grade persisting over 10- and 20-year follow-ups (Bloom & Field, 1971). This association has been confirmed by several other studies (Wolff, 1966; Hamlin, 1968; Champion *et al.*, 1972; Elston *et al.*, 1980) and Eichner *et al.* (1970) have shown good comparability between the Bloom method and the nuclear-grading method of Black *et al.* (1955). The results from this study provide further confirmation, based on a large number of patients, that the histological grade of a tumour has a highly significant influence on the prognosis of the patient (Fig. 5). It is difficult to make direct comparisons with Bloom & Richardson's study (1957) particularly as the overall survival at 5 years in our series is considerably higher (71% *vs.* 50%). However, in both series the 5-year survival of patients with Grade I tumours (CRC, 87%, Bloom & Richardson, 75%) is considerably better than those with Grade III tumours (CRC, 60%, Bloom & Richardson 32%). Although they account for only 12% of the invasive carcinomas, the fact that the survival curve for Grade I tumours is the same as for intraduct carcinomas, confirms the very good prognosis for patients with these tumours. It will be of interest to note whether this good prognosis is maintained when a full 10-year follow-up of all patients is obtained.

The term "medullary carcinoma" has been used to denote a particular type of breast cancer characterized by a circumscribed margin, relatively poor histological

FIG. 12.—Comparison of survival rates of patients with a severe cell reaction in a Grade III tumour and all patients with Grade II tumours ( $\chi^2 = 0 \cdot 37$, $P = 0 \cdot 54$).

FIG. 13.—Comparison of the survival rates of patients having a severe cell reaction and Grade II or III tumour with all other patients whose pathology has been assessed ( $\chi^2 = 0 \cdot 89$, $P = 0 \cdot 35$). The curve for patients having a severe cell reaction and Grade III tumour (Fig. 12) is superimposed.

differentiation and an intense lymphoplasmacytic infiltrate in the stroma. Despite the poor differentiation, this type of tumour has been reported to have a relatively favourable prognosis (Moore & Foote, 1949; Bloom *et al.*, 1970; Ridolfi *et al.*, 1977) and there has been speculation that the lymphoplasmacytic infiltrate

FIG. 14.—Comparison of survival according to treatment policy in patients with uninvolved nodes (−ve) ( $\chi^2=1\cdot88$, $P=0\cdot17$) and in patients with histologically involved nodes (+ve) ( $\chi^2=0\cdot24$, $P=0\cdot63$).

TABLE V.—*Comparison between WP and DXT local-recurrence-free rates (%) in patients with histologically assessed nodes*

| | | 5-year rate (%) ± s.e. | | | Logrank analysis | | | | | | |
|---|---|---|---|---|---|---|---|---|---|---|---|
| | | | | | WP | | DXT | | | | |
| | n | Overall | WP | DXT | O | E | O | E | HR | $\chi^2$ | $P$ |
| Node negative | (179) | 87·0±2·5 | 81·2±4·2 | 92·3±2·8 | 19 | 13·9 | 11 | 16·1 | 1·88 (0·9–4·0) | 3·55 | 0·06 |
| Node positive | (220) | 73·2±3·2 | 58·9±3·4 | 88·2±3·4 | 43 | 25·4 | 14 | 30·6 | 3·62 (2·0–6·7) | 20·1 | <0·001 |

represents an immunological host-defence mechanism (Berg, 1959). Ridolfi *et al.* (1977) showed a significantly better survival at 10 years for medullary carcinoma (84%) than for non-medullary carcinoma (63%) whilst Bloom *et al.* (1970) demonstrated persistence of this effect in a 20-year follow-up (medullary 48% survival, non-medullary 16%). It has also been shown that, when the intensity of the cell infiltrates is assessed in all the tumours in a series, the prognosis is better with the more severe reaction (Black *et al.*, 1955; Berg, 1959; Hamlin, 1968). In the CRC trial, an assessment of cell reaction was made in all cases of invasive carcinoma. For the purposes of analysis, the 4 degrees of infiltrate were combined to form 2 reaction groups, "mild" and "severe". The survival curves for both groups were identical (Fig. 9) indicating no advantage from severe reaction. In common with previous reports (Champion *et al.*, 1972; Blamey *et al.*, 1979) the severe infiltrates were usually associated with less differentiated tumours (Fig. 10), and the survival analysis may have been influenced by the inclusion in the mild-reaction group of well differentiated tumours with a more favourable prognosis. Accordingly, the effect of cell reaction was tested within each grade, and only in

Grade III tumours did patients in the severe-reaction group have a better survival (Fig. 11). A precise comparison with medullary carcinoma cannot be made in this study, as histological typing was not undertaken. The closest approximation to medullary carcinoma is the subgroup composed of tumours of Grade II and III exhibiting a severe cell reaction; patients in this group did not have a better survival than other patients in the study (Fig. 13). This casts doubt on the contention that it is the lymphoplasmacytic infiltrate which is responsible for the relatively favourable prognosis of medullary carcinoma (Berg, 1959). In this study the only predictive value of a severe cell reaction lies in the fact that its presence in a Grade III tumour improves the survival curve to that of the overall Grade II group (Fig. 12). These results suggest, in common with some previous reports (Wolff, 1966; Blamey *et al.*, 1979) that assessment of cell reaction provides limited prognostic information.

The relationship between regional lymph-node metastasis and prognosis in breast cancer is well established (Cutler *et al.*, 1969; Fisher *et al.*, 1975). Although the removal of axillary lymph nodes was discouraged, nodes were removed incidentally in 27% of cases. The survival curves confirm the significantly poorer prognosis of patients with histologically involved axillary nodes (Fig. 14).

The most important point to emerge from follow-up in the tenth year of this trial is that, although there is no difference in survival between patients in the 2 treatment groups, those in the DXT group had fewer local recurrences (CRC Working Party, 1980*a*). This poses a therapeutic dilemma. Should all patients undergoing simple mastectomy receive immediate post-operative radiotherapy to spare some 20–30% the anxiety of recognizing early failure of treatment, or could delayed radiotherapy eventually achieve the same degree of local control and thus spare most women unnecessary prophylactic radiotherapy? Consideration of the pathological data clarifies this problem. Local recurrence rates are significantly higher in the WP group for patients with Grade II and III tumours, but not Grade I tumours (Figs 7 and 8, Table III) and for patients with histologically involved nodes (Table V). Furthermore, within Grades II and III the risk tends to increase with increasing tumour size (Table III). Immediate post-operative radiotherapy could certainly be recommended for patients with large Grade II or III tumours and histologically involved lymph nodes, whilst patients with small Grade I tumours and histologically negative nodes may not require adjuvant radiotherapy. Thus, for the first time, data are presented that may allow the clinician to select the extent of *local* therapy on the basis of histopathological criteria.

The purpose of the pathological part of the trial was to test certain established and potential pathological factors for prognostic significance. The results to date indicate that tumour size, histological grade and lymph-node metastasis provide significant markers of survival in breast cancer. This is in close agreement with the prospective study of Blamey *et al.* (1979) who have used these factors to devise an index of poor prognosis. They can also be used to determine local treatment policy. In planning the next generation of trials of adjuvant therapy, these factors should play an important part in the stratification of patients and therapy.

We wish to acknowledge our debt to the late Mr B. M. Truscott, surgeon at Addenbrooke's Hospital, Cambridge, as the trial was largely due to his initiative. We thank the 182 surgeons, 53 radiotherapists, and 82 pathologists who deserve most of the credit for the successful recruitment to the study; the Cancer Research Campaign Fellows who in their turn helped with the administration of the study: Professor C. J. Magarey, Mr M. H. Edwards, Mr D. Sumner, Mr J. A. Simpson, Mr A. M. MacDonald, and Mr D. J. Leaper; and Dr K. MacRae and the late Dr G. Edelstyn, who were former members of the working party of this study. Mr R. Peto provided invaluable advice on statistical methodology and Miss J. Crabtree, who was the original secretary to this project, has been pleased to hand over the typing of the innumerable drafts of this paper to Ms J. Reeley. Finally we wish once more to acknowledge the very general financial support of the Cancer Research Campaign.

### REFERENCES

Baum, M., Edwards, M. H. & Margarey, C. J. (1972) Organisation of clinical trial on national scale: Management of early cancer of the breast. *Br. Med. J.*, iv, 476.

Berg, J. W. (1959) Inflammation and prognosis in breast cancer. *Cancer*, **12**, 714.

Black, M. M., Opler, S. R. & Speer, F. D. (1955) Survival in breast cancer cases in relation to the structure of the primary tumor and regional lymph nodes. *Surg. Gynecol. Obstet.*, **100**, 543.

Blamey, R. W., Davies, R. J., Elston, C. W., Johnson, J., Haybittle, J. L. & Maynard, P. V. (1979) Prognostic factors in breast cancer: The formation of a prognostic index. *Clin. Oncol.*, **5**, 227.

Bloom, H. J. G. (1950*a*) Prognosis in carcinoma of the breast. *Br. J. Cancer*, **4**, 259.

Bloom, H. J. G. (1950*b*) Further studies on prognosis of breast carcinoma. *Br. J. Cancer*, **4**, 347.

Bloom, H. J. G. (1962) The role of histological grading in the study of breast cancer. *Acta Un. Int. Cancer*, **18**, 843.

Bloom, H. J. G. & Field, J. R. (1971) Impact of tumour grade and host resistance on survival of women with breast cancer. *Cancer*, **28**, 1580.

Bloom, H. J. G. & Richardson, W. W. (1957) Histological grading and prognosis in breast cancer: A study of 1409 cases of which 359 have been followed for 15 years. *Br. J. Cancer*, **11**, 359.

Bloom, H. J. G., Richardson, W. W. & Field, J. R. (1970) Host resistance and survival in carcinoma of breast: A study of 104 cases of medullary carcinoma in a series of 1,411 cases of breast cancer followed for 20 years. *Br. Med. J.*, iii, 181.

Cancer Research Campaign Working Party (1976) Management of early cancer of the breast. Report on an international multicentre trial supported by the Cancer Research Campaign. *Br. Med. J.*, i, 1035.

Cancer Research Campaign Working Party (1980*a*) Cancer Research Campaign (King's/Cambridge) trial for early breast cancer. A detailed update at the tenth year. *Lancet*, i, 55.

Cancer Research Campaign Working Party (1980*b*) Trials and tribulations: Thoughts on the organisation of multicentre clinical studies. *Br. Med. J.* **280** 918.

Champion H. R., Wallace, I. W. J. & Prescott, R. J. (1972) Histology in breast cancer prognosis. *Br. J. Cancer*, **26**, 129.

Cutler, W. J., Black, M. M., Mork, T., Harvei, S. & Freeman, C. (1969) Further observations on prognostic factors in cancer of the female breast. *Cancer*, **24**, 653.

Eichner, W. J., Lemon, H. M. & Friedell, G. (1970) Tumor grade in the prognosis of breast cancer. *Nebraska Med. J.*, **55**, 405.

Elston, C. W., Blamey, R. W., Johnson, J., Bishop, H. M., Haybittle, J. L. & Griffiths, K. (1980) The relationship of oestradiol receptor (ER) and histological tumour differentiation with prognosis in human primary breast carcinoma. In *Breast cancer—Experimental and Clinical Aspects.* (Eds Mouridsen & Palshof). New York: Pergamon Press. p. 59.

Fisher, B., Slack, N. H., Bross, I. D. J. & co-operating investigators (1969) Cancer of the breast Size of neoplasm and prognosis. *Cancer*, **24**, 1071.

Fisher, B., Slack, N., Katrych, D. & Wolmark, N. (1975) Ten year follow-up results of patients with carcinoma of the breast in a co-operative clinical trial evaluating surgical adjuvant chemotherapy. *Surg. Gynecol. Obstet.*, **140**, 528.

Greenough, R. B. (1925) Varying degrees of malignancy in cancer of the breast. *J. Cancer Res.*, **9**, 453.

Hamlin, I. M. E. (1968) Possible host resistance in carcinoma of the breast: A histological study. *Br. J. Cancer*, **22**, 383.

Moore, O. S. & Foote, F. W. (1949) The relatively favourable prognosis of medullary carcinoma of the breast. *Cancer*, **2**, 635.

Patey, D. H. & Scarff, R. W. (1928) The position of histology in the prognosis of carcinoma of the breast. *Lancet*, i, 801.

Peto, R., Pike, M. C., Armitage, P. & 6 others (1977) Design and analysis of randomized clinical trials requiring prolonged observation of each patient—II Analysis and examples. *Br. J. Cancer*, **35**, 1.

Ridolfi, R. L., Rosen, P. P., Port, A., Kinne, D. & Miké, V. (1977) Medullary carcinoma of the breast: A clinicopathologic study with 10 year follow-up. *Cancer*, **40**, 1365.

Wolff, B. (1966) Histological grading in carcinoma of breast. *Br. J. Cancer*, **20**, 36.